# Opsoclonus-Myoclonus-Ataxia Syndrome: A Rare Outcome Following Routine Vaccinations

**DOI:** 10.7759/cureus.74413

**Published:** 2024-11-25

**Authors:** Catarina Leuzinger-Dias, Tomás Ferrão, Sandra Rebimbas, Filipe Palavra, Joana Amaral

**Affiliations:** 1 Neuropediatrics, Centre for Child Development, Hospital Pediátrico, Unidade Local de Saúde de Coimbra, Coimbra, PRT; 2 Pediatrics, Unidade Local de Saúde da Região de Aveiro, Aveiro, PRT; 3 Neuropediatrics, Coimbra Institute for Clinical and Biomedical Research (iCBR) Faculty of Medicine, University of Coimbra, Coimbra, PRT

**Keywords:** myoclonus, neuropediatrics, opsoclonus-myoclonus-ataxia, pediatrics, vaccines

## Abstract

Opsoclonus-myoclonus-ataxia syndrome (OMAS) is a rare neurological disorder, affecting approximately 0.18 per million individuals annually. It presents with a triad of opsoclonus, myoclonus, and ataxia, often including cognitive dysfunction and behavioral disturbances. Even though OMAS is non-fatal, it frequently follows a chronic-relapsing course, resulting in long-term neuropsychological sequelae in over 50% of patients. Diagnosis is challenging due to variable symptom presentation and the lack of specific diagnostic markers.

We report a case of a two-month-old infant who developed OMAS after routine vaccinations, a rare post-vaccination occurrence. Initial treatment with high-dose corticosteroids successfully resolved symptoms, but the patient experienced a relapse during medication tapering. The relapse occurred following another vaccination, suggesting a potential link between the immune response triggered by vaccination and OMAS symptoms. Despite the early relapse, the patient had a favorable recovery, with normal neurodevelopment at 24 months and no further relapses. This case raises awareness of the rare autoimmune post-vaccine reaction as a possible etiology of OMAS, which is infrequently reported in the literature. With this report, we aimed to underscore the importance of early recognition of OMAS, particularly in infants, and highlight the potential for a positive outcome with timely diagnosis and treatment.

## Introduction

Opsoclonus-myoclonus-ataxia syndrome (OMAS), also known as Kinsbourne syndrome is a rare neurological disorder, with an estimated annual incidence of 0.18 per million individuals [1]. OMAS is a complex autoimmune neurological disorder, typically affecting toddlers, with a mean age of onset of 1.5-2 years, although it can manifest as early as three months or in adult life [1,2]. Some studies suggest a higher prevalence in girls, consistent with the pattern seen in other autoimmune diseases [3].

The syndrome gets its name and is defined by a characteristic clinical triad as follows: opsoclonus, an involuntary, high frequency, and multidirectional ocular saccadic movements [4]; myoclonus, consisting of sudden jerk movements caused by involuntary muscle contractions [5]; and cerebellar ataxia. These are also often accompanied by cognitive dysfunction, behavioral disturbances, irritability, or sleep disruption, which are most prominent during the acute phase or in cases of relapse [2].

Symptoms at onset can vary significantly in their expression severity and are not necessarily all present simultaneously [6]. Onset often begins with behavioral symptoms, such as excessive irritability, sleep disturbances, developmental stagnation, or regression, followed by subacute onset of ataxia, which can take days to weeks to install [2]. Myoclonus in the limbs and trunk, along with hypotonia, commonly emerges [7]. Opsoclonus, while a hallmark of the syndrome, may be intermittent or late in onset, sometimes weeks after the onset of ataxia contributing to diagnostic challenges [5]. Due to the variable presentation and the absence of one or more distinctive features in the early stages of the disease, diagnosis can be delayed by weeks or even months and OMAS should be considered even when only some of the clinical features are present [8].

In terms of etiology, OMAS is often classified as paraneoplastic, with around 50% of pediatric cases linked to neuroblastoma, and a smaller percentage to other tumors, such as ovarian teratoma or hepatoblastoma [2,8]. The remaining cases can be parainfectious or related to other systemic diseases, medications, or toxic agents [9]. In many cases, however, no clear cause is identified after extensive investigations, and the condition is classified as idiopathic. Viral prodrome is common and OMAS has been reported following vaccination in the month preceding symptom onset [10]. Diagnosis is primarily clinical, as no specific laboratory tests definitively confirm OMAS. However, investigations should include screening for neuroblastoma [2].

Given the autoimmune nature of the syndrome, treatment usually involves immunotherapy, mainly with corticosteroids, adrenocorticotropic hormone, or intravenous immunoglobulin (IVIG) [9,11]. While some cases present acutely and improve with treatment, a significant proportion of children may have clinical relapses, often triggered by intercurrent illness or tapering of immunosuppressive therapy [12]. Relapsing cases tend to result in long-term neurological sequelae, mostly learning disabilities and behavioral issues [5].

We present a rare case of OMAS related to vaccination in a young child, aiming to raise awareness of its presentation and unique aspects in early childhood.

## Case presentation

The patient is a female infant, born to a healthy, non-consanguineous couple from an uneventful first pregnancy. She was delivered via cesarean section at 39 weeks gestation, with an uncomplicated transition to extra-uterine life. Her birth weight was 2300 g, placing her below the 10th percentile on the Fenton growth chart. She underwent 24 h of phototherapy to treat neonatal jaundice and was discharged four days after birth. The following weeks were uneventful, with the patient receiving mixed breastfeeding and iron supplementation.

At two months and 14 days, she presented to the emergency department with 6 h onset of irritability and episodes of brief, erratic multidirectional ocular movements occurring every 5-10 min. These episodes were also associated with oromandibular clonus. The patient remained alert and conscious between episodes, though irritable. No other abnormal movements were noted. She was afebrile and without any other symptoms.

Regarding her recent history, the patient had received the meningococcal B vaccine three days before the onset of symptoms and the 13-valent pneumococcal and hexavalent vaccines (tetanus, diphtheria, pertussis, poliomyelitis, hepatitis B, and *Haemophilus influenzae*) 11 days prior, in accordance with the national vaccination program schedule. Additionally, both parents had experienced gastrointestinal symptoms for the previous two days.

**Figure 1:**
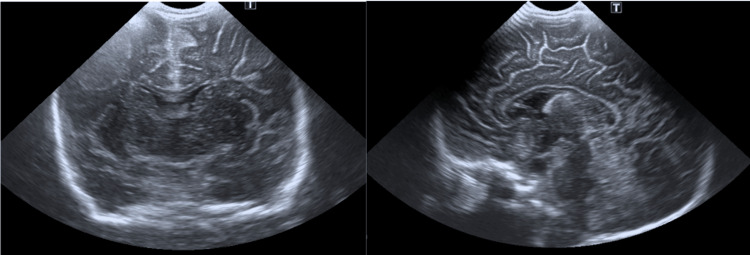
Transfontanellar ultrasound image - third coronal plane (left) and median sagittal plane (right).

On physical examination, she was tachycardic, normotensive, afebrile, and eupneic, with a normotensive fontanelle. Between episodes, she remained alert and active, with no focal neurological deficits, maintaining eye contact and reacting to auditory stimuli, but refused to feed. Preliminary investigation revealed lymphocytosis (9830/µL) and thrombocytosis (801000/µL) but was otherwise unremarkable (Table 1), including a normal transfontanellar ultrasound (Figure 1).

**Table 1:** Patients' preliminary laboratory findings at admission in the emergency department.

Test	Patient result	Normal range
Leukocytes	14500/µL	5500-18000/µL
Neutrophils	3370/µL	1000-8500/µL
Lymphocytes	9830/µL	4000-10500/µL
Hemoglobin	12.5 g/dL	9.5-13.5 g/dL
Platelets	801000/µL	150000-450000/µL
Erythrocyte sedimentation rate	18 mm/h	1-20 mm/h
Urea	17 mg/dL	4-19 mg/dL
Serum creatinine	0.33 mg/dL	0.32-0.53 mg/dL
Sodium	136 mmol/L	135-145 mmol/L
Potassium	5 mmol/L	3.5-5.0 mmol/L
Chloride	107 mmol/L	101-109 mmol/L
Calcium (total)	9.5 mg/dL	8.6-9.7 mg/dL
Magnesium	2.1 mg/dL	1.5-2.2 mg/dL
Phosphates	5 mg/dL	4.8-8.4 mg/dL
Bicarbonate	23.1 mmol/L	22-26 mmol/L
pCO_2_	35 mmHg	35-45 mmHg
Lactate	2 mmol/L	0-2 mmol/L
C-reactive protein	0.2 mg/dL	<0.5 mg/dL
Procalcitonin	0.02 ng/mL	<0.5 ng/mL
Serum glucose	102 mg/dL	74-106 mg/dL
Aspartate aminotransferase	35 U/L	20-67 U/L
Alanine aminotransferase	22 U/L	5-33 U/L
Alkaline phosphatase	317 U/L	134-518 U/L
Gamma-glutamyl transferase	31 U/L	8-127 U/L
Lactate dehydrogenase	268 U/L	163-453 U/L
Urinary vanillylmandelic acid/creatinine ratio	16.4 mg/g	<25 mg/g
Neuroenolase	14 ng/mL	<15 ng/mL

**Figure 2:**
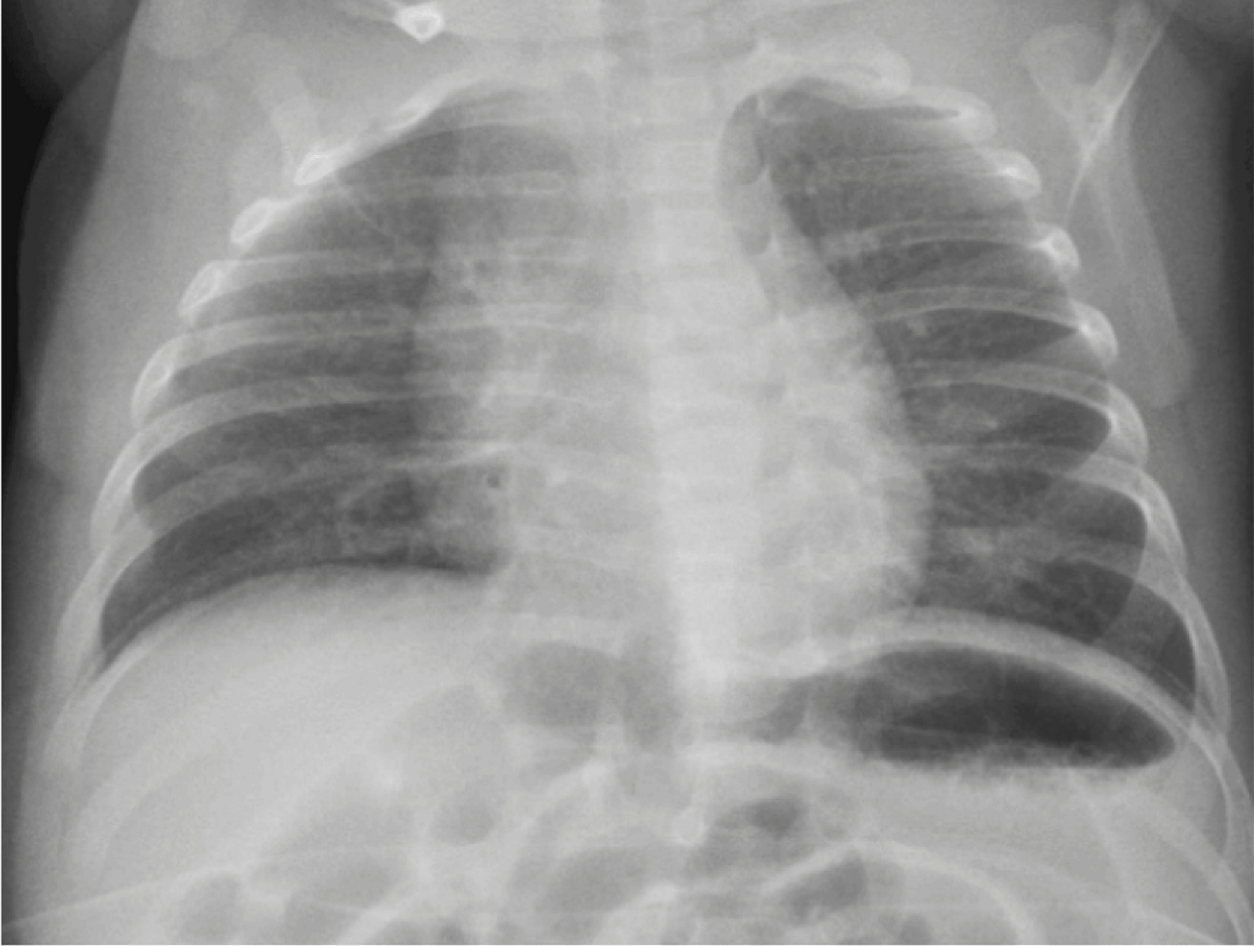
Chest x-ray showing a visible thymus with no mediastinal widening.

**Figure 3:**
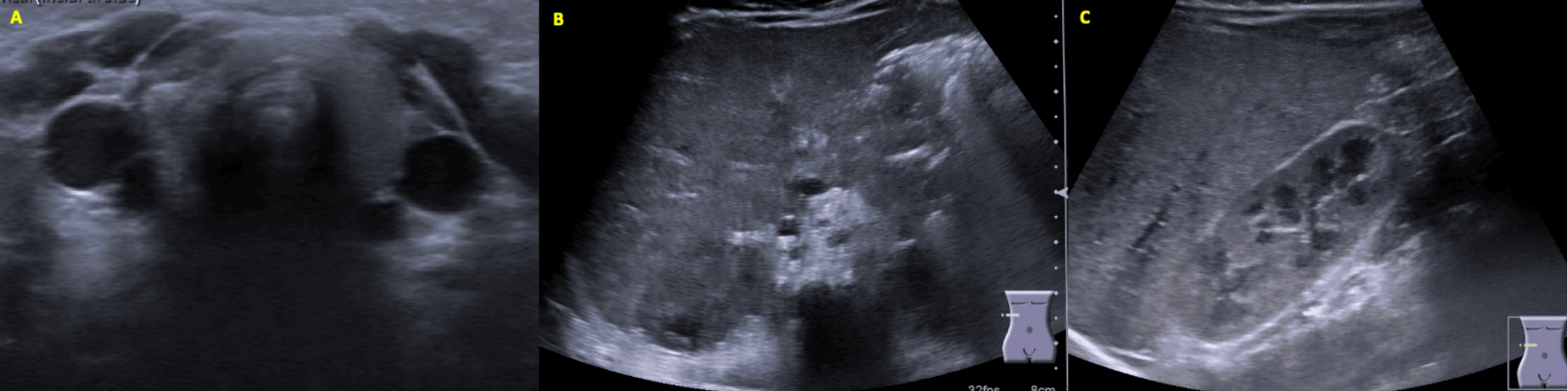
Serial images of thyroid (A), hepatic (B), and renal (C) ultrasound.

**Figure 4:**
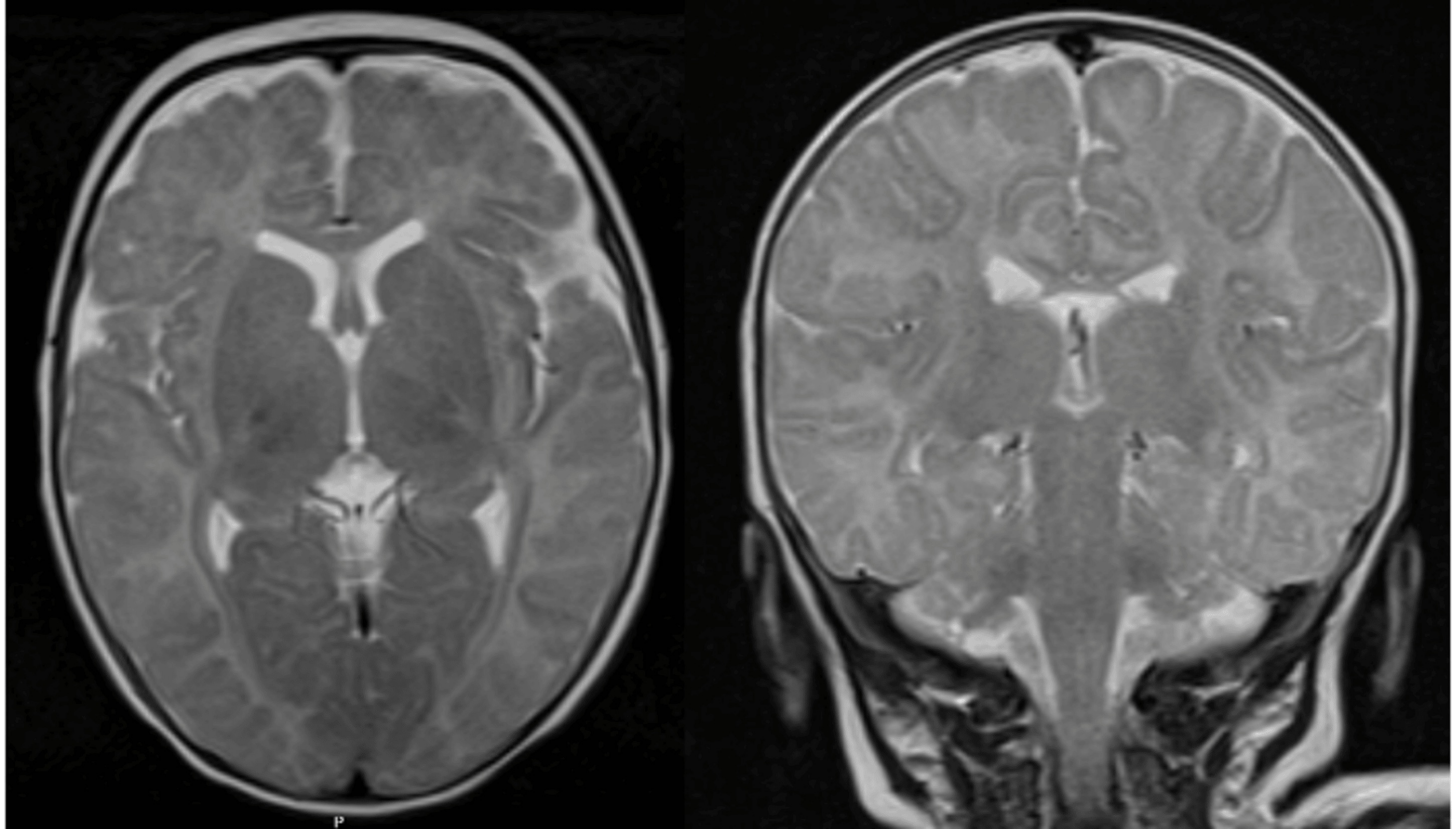
T2-weighted brain MRI - axial (left) and coronal (right).

The patient was transferred to a tertiary care hospital for evaluation by a pediatric neurologist. She maintained episodes of the described symptoms, but neurological examination between episodes was normal, with a normotensive and pulsatile fontanelle, normal myotatic reflexes, and no focal deficits. OMAS was suspected, and she was admitted for further workup. Imaging studies, including a chest x-ray, and cervical, abdominal, and pelvic ultrasounds, did not reveal any masses suggestive of neuroblastoma (Figures 2, 3A-3C). Urinary vanillylmandelic acid (VMA) levels were within normal ranges (Table 1). Ophthalmologic evaluation was normal, and electroencephalography (EEG) showed no abnormal electrical activity. Cranial MRI findings were unremarkable (Figure 4).

On the third day of hospitalization, a five-day course of high-dose intravenous methylprednisolone (30 mg/kg/day) was initiated, resulting in a significant improvement of symptoms and resolution of the opsoclonus. After 11 days of hospitalization, the patient was discharged asymptomatic, with instructions to continue oral prednisolone on a tapering schedule. Nevertheless, 48 h after discharge, following a reduction in prednisolone to 1 mg/kg, she returned to the emergency department with a recurrence of erratic eye movements. The steroid dosage was increased to 2 mg/kg, and after 24 h of observation, she had a favorable response and was discharged again with instructions to maintain the increased steroid dosage for two weeks.

One month after the initial episode, the patient was readmitted for recurrent opsoclonus. She had developed rhinorrhea a few days prior, without fever or cough, and had received an anti-meningococcal vaccine two days before the relapse. Nasopharyngeal swab analysis via polymerase chain reaction (PCR) detected parainfluenza virus type 3, but other laboratory results were unremarkable.

An extensive workup was repeated, including VMA levels, neuroenolase, and abdominal ultrasound which remained normal, ruling out neuroblastoma. Opsoclonus episodes diminished, and it was hypothesized that vaccination had triggered the relapses. The patient was discharged after four days with a plan to transiently increase steroid dosages during future vaccination periods.

Steroid therapy was successfully discontinued at five months of age, and the patient has remained episode-free since, with no further relapses after following vaccines. Neuroenolase levels assessed during follow-up were negative. The patient is currently 24 months old with normal neurodevelopment, sleep, and behavioral patterns.

## Discussion

OMAS is a rare syndrome with a wide range of clinical presentations, and not all patients exhibit the full triad of symptoms (it is worth noting the absence of ataxic semiology in our case, although the child's age must also be taken into account). This variability, in association with the absence of a specific diagnostic marker, further contributes to the delay in the diagnosis. In this case, early observation by a pediatric neurologist on the first day of symptoms enabled a timely diagnosis, based solely on clinical aspects.

Despite not having an analytical hallmark of the disease, children with OMAS display functionally active auto-antibodies, with elevated T and B cells and other neuroinflammatory elements, such as oligoclonal bands in cerebrospinal fluid [13]. This immune dysregulation along with other pro-inflammatory changes in the cytokine network supports the hypothesis of an underlying auto-immune phenomenon [14]. As a result, immunomodulatory therapies have been the cornerstone of OMAS treatment, with corticosteroids being the first line of therapy, often for extended periods.

In our patient’s case, immunotherapy was started after neuroblastoma was excluded. Given that nearly half of all pediatric cases are associated with this neoplasm, a thorough assessment for underlying malignancy is a crucial aspect of OMS management [14].

First-line treatment consists of high-dose corticosteroids, followed by a lower maintenance dose after symptoms have resolved. The goal was to improve neurological symptoms, as well as to address potential neuropsychological disturbances, although such disturbances were less apparent in our patient due to her young age [15].

Unfortunately, tapering off corticosteroids often leads to relapse, and many neuropsychological symptoms have a suboptimal response to treatment [15]. Our patient first relapsed when therapy was weaned, which is in accordance with the study by Brunklaus et al., where 82% of patients relapsed during steroid weaning [10].

After a favorable response to a therapeutic adjustment, her symptoms remerged two weeks later following vaccination. The same had happened during her initial OMAS episode when she received eight vaccines within two weeks before the onset of neurological symptoms. Considering that humoral immune mechanisms seem to play a role in OMAS, it is possible that vaccination, either alone or in combination with mild viral infection, generated an immune response that led to antibody-mediated neuronal dysfunction.

Considering that humoral immune mechanisms seem to play a role in OMAS, it is possible that vaccination, either alone or in combination with mild viral infection, generated an immune response that led to antibody-mediated neuronal dysfunction.

Thus, we concluded that our patient had idiopathic OMAS triggered by vaccination, which is rare, but there are reports of OMAS after human papillomavirus [16], Rubella [17], and flu vaccinations [18]. Brunklaus et al. also reported that 8% of OMAS cases had received vaccinations within one month before symptom onset [10]. Regarding treatment course, while some patients are dependent on steroids, our patient was able to discontinue treatment after two months, with no further relapses [19]. 

Despite our patient’s good results, OMAS outcomes over time have historically been poor. Many studies report long-term impairment, with more than half of cases with learning disabilities and behavioral difficulties, even though there is an improvement in neurological symptoms [10].

In a large study by Brunklaus et al., which reviewed 101 childhood OMAS cases over 53 years, 60% of patients were found to have residual motor problems, while two-thirds had speech impairments [10]. Additionally, 51% experienced cognitive deficits, and 46% had chronic behavioral issues. Only one-third of the patients achieved a normal neuropsychological outcome. Thus, continuous follow-up and close surveillance for neurodevelopment sequelae are essential in these cases.

There is still debate regarding prognostic factors in OMAS. Relapses are frequent, and cognitive and behavioral disturbances became a larger part of the clinical course [5]. Some studies suggest that the severity of initial symptoms correlates with a chronic-relapsing disease course and future learning disabilities and that younger age at onset is linked to more severe cognitive impairment [11]. Children with a chronic-relapsing course, tend to experience the most severe cognitive deficits, whereas those with a monophasic course have a more benign prognosis [19]. Additionally, some studies suggest that early initiation of treatment improves outcomes [20], but this is not consistent in the literature, as others report that neither early nor intensive treatment significantly prevents poor outcomes [15,19].

Throughout our patient’s follow-up, repeated neurological assessments and laboratory evaluations were conducted to rule out neuroblastoma. There is no consensus on how long to continue screening for underlying malignancies, and this decision often lies on clinical judgment. Nonetheless, these reevaluations are essential, given that some OMAS cases occur months before tumor development, meaning negative initial investigation does not entirely rule out malignancy. At 24 months of age, our patient remains asymptomatic and out of treatment, with an uneventful neurodevelopment, reaching all developmental milestones at key ages.

## Conclusions

Opsoclonus-myoclonus-ataxia syndrome (OMAS) is a rare neurological disorder that can lead to severe neuropsychological sequelae in over 50% of patients and may follow a chronic-relapsing course, difficult to manage. We present a case of a two-month-old infant who developed OMAS after vaccinations, a rare occurrence in the literature, and who had a good neuropsychological evolution, despite having relapsed during the initial tapering of medication. This case highlights the importance of early recognition of neurological symptoms in infants and the potential for a positive outcome with prompt diagnosis and treatment.
